# Intrauterine Growth Restriction: Need to Improve Diagnostic Accuracy and Evidence for a Key Role of Oxidative Stress in Neonatal and Long-Term Sequelae

**DOI:** 10.3390/cells13060501

**Published:** 2024-03-13

**Authors:** Eva Nüsken, Sarah Appel, Leon Saschin, Celien Kuiper-Makris, Laura Oberholz, Charlotte Schömig, Anne Tauscher, Jörg Dötsch, Angela Kribs, Miguel A. Alejandre Alcazar, Kai-Dietrich Nüsken

**Affiliations:** 1 Clinic and Polyclinic for Pediatric and Adolescent Medicine, University Hospital Cologne, Faculty of Medicine, University of Cologne, 50937 Cologne, Germany; eva.nuesken@uk-koeln.de (E.N.);; 2 Department of Obstetrics and Gynecology, University Hospital Leipzig, 04103 Leipzig, Germany; 3 Institute for Lung Health (ILH), University of Giessen and Marburg Lung Center (UGMLC) and Cardiopulmonary Institute (CPI), Member of the German Center for Lung Research (DZL), 35392 Giessen, Germany; 4 Center for Molecular Medicine Cologne (CMMC), University of Cologne, 50931 Cologne, Germany; 5 Cologne Excellence Cluster on Cellular Stress Responses in Aging-Associated Diseases (CECAD), University of Cologne, 50931 Cologne, Germany

**Keywords:** intrauterine growth restriction, fetal growth restriction, acute and long-term sequelae, oxidative stress, antioxidants

## Abstract

Intrauterine growth restriction (IUGR) and being small for gestational age (SGA) are two distinct conditions with different implications for short- and long-term child development. SGA is present if the estimated fetal or birth weight is below the tenth percentile. IUGR can be identified by additional abnormalities (pathological Doppler sonography, oligohydramnion, lack of growth in the interval, estimated weight below the third percentile) and can also be present in fetuses and neonates with weights above the tenth percentile. There is a need to differentiate between IUGR and SGA whenever possible, as IUGR in particular is associated with greater perinatal morbidity, prematurity and mortality, as well as an increased risk for diseases in later life. Recognizing fetuses and newborns being “at risk” in order to monitor them accordingly and deliver them in good time, as well as to provide adequate follow up care to ameliorate adverse sequelae is still challenging. This review article discusses approaches to differentiate IUGR from SGA and further increase diagnostic accuracy. Since adverse prenatal influences increase but individually optimized further child development decreases the risk of later diseases, we also discuss the need for interdisciplinary follow-up strategies during childhood. Moreover, we present current concepts of pathophysiology, with a focus on oxidative stress and consecutive inflammatory and metabolic changes as key molecular mechanisms of adverse sequelae, and look at future scientific opportunities and challenges. Most importantly, awareness needs to be raised that pre- and postnatal care of IUGR neonates should be regarded as a continuum.

## 1. Introduction

Since the first epidemiological associations between low birth weight and elevated risk for coronary heart disease [1], it has become widely accepted that the underlying clinical condition and its molecular basis strongly affect the frequency and severity of potential neonatal complications and determine the risk for long-term morbidity, rather than birth weight itself [2,3,4]. Most importantly, intrauterine growth restriction (IUGR) and being small for gestational age (SGA) are two distinct conditions [5,6,7] with different implications for child development.

While being born SGA below the tenth percentile may be physiological (e.g., when both parents are small), IUGR indicates a pathophysiology during pregnancy [8]. According to the international Delphi consensus, the diagnosis of IUGR in fetuses below the tenth percentile of estimated fetal weight (EFW) and/or abdominal circumference (AC) should take both decelerating fetal growth trajectories and abnormal Doppler findings into account [7]. Further important aspects are that IUGR may also have occurred in neonates with birth weights on or above the tenth percentile [5,9] and may be missed at a birth weight ≥ third and < tenth percentile and normal Doppler findings [10]. In most of these cases, intrauterine growth trajectories are not taken into account or cannot be assessed due to missing data during pregnancy. Sometimes, inappropriately slow growth cannot be identified by any of the standard biophysical diagnostic tools [10].

Thus, even in highly developed health care systems, the diagnosis of IUGR/exclusion of IUGR in SGA is not always made. Epidemiologic and clinical studies with strict IUGR criteria are rare. Above all, there is a persistent gap in knowledge of which sequelae are truly and irreversibly related to IUGR and which sequelae might be counteracted by, e.g., optimized early life conditions.

Our review article presents advances in increasing the diagnostic accuracy of IUGR and highlights the interrelationship between IUGR and clinically relevant short- and long-term sequelae, with the aim to reliably identify children being “at risk”. We also summarize current knowledge on the role of oxidative stress and consecutive inflammatory and metabolic changes as a molecular key to adverse sequelae, and look at related future scientific opportunities and challenges. Based on the above, we discuss approaches to improve the prevention and aftercare of IUGR.

## 2. Inconsistent Diagnostic Criteria of Intrauterine Growth Restriction

IUGR, in recent publications mostly referred to as fetal growth restriction (FGR) [10,11], is reliably diagnosed by multiple measurements of fetal size over time only [10].

**Figure 1:**
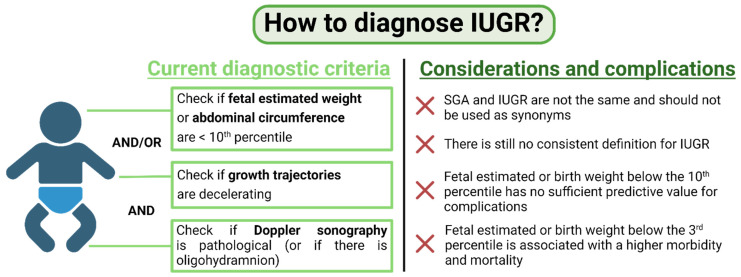
How to diagnose IUGR? Current diagnostic criteria for IUGR are shown on the left. Considerations and complications in the diagnosis are shown on the right. Figure created with BioRender.com.

When multiple measurements of fetal size are not available, abnormal Doppler findings define whether SGA fetuses below the tenth percentile should be diagnosed as IUGR, according to the international Delphi consensus (Figure 1) [7]. Nevertheless, some guidelines also still use the simple presence of EFW or abdominal circumference (AC) below the tenth percentile of gestational age as sufficient for the diagnosis of IUGR [11]. Below the third percentile, there is international agreement that the fetuses should be defined as IUGR even if there is no further evidence of growth crossing percentiles or impaired placental function [7]. This compromise is based on the fact that below the third percentile, the risk of stillbirth and neonatal morbidity is comparable to fetuses with proven Doppler abnormalities [7].

In a large cohort study, the combined use of fetal growth trajectories and EFW increased the discrimination rate between fetuses “at risk” for neonatal morbidity and low-risk low-EFW fetuses [12]. Interestingly, IUGR alone without low EFW was not associated with neonatal morbidity in this study [12]. However, another study was also able to associate IUGR without low EFW with an increased risk of stillbirths when using different models to define slow fetal growth [13]. To address these apparent discrepancies and further improve the risk assessment for neonatal and long-term sequelae, the onset of IUGR during pregnancy, the presence of Doppler anomalies in the umbilical and middle cerebral arteries, as well as short-term variation (STV) in computerized analysis of CTG (cCTG) and Ductus venosus Doppler anomalies have been validated as additional diagnostic criteria [7,14]. Despite these advances, guidelines for the diagnosis of IUGR focus mainly on intrauterine or early postnatal morbidities and complications but do not sufficiently consider long-term sequelae.

## 3. Approaches to Increase Diagnostic Accuracy and Identify Neonates Being “at Risk”

A clear definition and diagnosis of IUGR is a prerequisite to reliably identify neonates being “at risk” for perinatal and long-term complications. Disruptive factors such as pre-eclampsia, gestational diabetes, chromosomal disorders, infections, etc., must also be correctly diagnosed and appropriately taken into account. However, as mentioned above, many problems can arise when diagnosing IUGR under everyday conditions, ranging from a lack of data to lack of resources to diagnostic challenges. For instance, late-onset IUGR (≥32 weeks of gestation) is much more difficult to detect than early-onset IUGR (< 32 weeks of gestation) because the umbilical artery Doppler is mostly normal [15]. Some IUGR cases remain completely undetected during pregnancy despite sufficient prenatal examinations, especially when the fetus is not absolutely growth-restricted within the population based reference chart, but only in relation to its individual growth potential [16]. To address this issue, a couple of promising studies have been published that have validated diagnostic criteria shortly before birth or perinatally.

### 3.1. Imaging Studies

Recent studies highlighted that novel Doppler variable combinations such as the cerebral–placental–uterine ratio using the pulsatility indices (PIs) of the middle cerebral artery, the umbilical artery, and the uterine arteries, have a higher predictive accuracy for identifying IUGR than Doppler measurements of only the umbilical artery [17]. The presence of retrograde blood flow in the aortic isthmus may be an additional indication of an increased risk of perinatal death, stillbirth, and respiratory distress syndrome in IUGR patients [18]. In late-onset IUGR, abnormal cerebroplacental ratio (CPR, i.e., the PI of the middle cerebral artery divided by PI of the umbilical artery) indicates the risk of hypoxemia more sensitively than the PIs of the middle cerebral artery or umbilical artery alone. Moreover, an abnormal cerebroplacental ratio significantly reflects maternal, fetal, and composite vascular malperfusion lesions [19]. Furthermore, umbilical vein blood flow (UVBF) is reduced in IUGR fetuses [20,21,22]. In late-onset IUGR, the Z-score of UVBF normalized for fetal abdominal circumference (UVBF/AC Z-score) predicted an adverse perinatal outcome more precisely than a mean uterine artery PI Z-score or CPR Z-score [23]. UVBF normalized by fetal weight is also useful to differentiate IUGR term fetuses “at risk” from SGA fetuses [24,25]. Maternal hemodynamic indices also may provide useful additional information. Maternal cardiac output, left ventricular mass, and peripheral vascular resistance are associated with EFW [26] and help to differentiate between IUGR and SGA [25].

Fetal magnetic resonance imaging (f-MRI) is time-consuming and requires many resources, but it is safe for the fetus and can also add important data to differentiate between IUGR and SGA [27]. Placental perfusion can be quantitatively assessed by Intravoxel Incoherent Motion (IVIM) MRI. The perfusion fraction of the placenta is significantly lower in IUGR compared to control placentas [28]. Diffusion-weighted MRI is able to identify microstructural alterations in IUGR placentas in vivo [29]. Circulatory redistribution can also be comprehensively assessed by MRI, which may be useful especially in late-onset IUGR [30].

### 3.2. Placental Work-Up

Unfortunately, histological examination of the placenta after birth is too rarely performed in everyday clinical practice, most likely due to a lack of resources. Postnatal placenta analysis offers the possibility of recognizing cases of IUGR at the time of birth, which may have remained undetected during pregnancy. Indeed, children classified as SGA based on normal fetal Doppler findings may show histological signs of placental underperfusion, suggesting intrauterine pathology [31]. Thus, the examination of the placenta can help to ensure that the child can be optimally cared for after birth. By assessing the gross morphology of the placenta, such as shape and size, conclusions can be drawn about the functionality of the organ. Studies have demonstrated a correlation between IUGR and decreased placental weight, diameter, or volume, which in sum contribute to decreased placental surface area [32,33]. Moreover, a reduction in surface area has been described histologically [34]. An overview about antenatal and postnatal placental findings indicative of IUGR is given in Salavati et al. [16].

In addition to morphological analyses of the placenta, biomarkers and molecular signatures from the placenta and amniotic fluid can help to distinguish between healthy pregnancies and pregnancies with IUGR. In an interesting study from 2022 [35], placental samples collected after delivery were analyzed using a metabolomics approach. The authors detected 220 metabolites, of which the concentrations of 179 metabolites were significantly altered in FGR placentas compared with control placentas. In total, 98.3% of the dysregulated metabolites showed lower concentrations in placenta samples from IUGR pregnancies. Placental 3-hydroxybutyric acid, glycine, and phosphatidylcholine with the C42:0 acyl-alkyl radical proved to be the best metabolites for differentiating between IUGR and control placenta samples. Although detection of IUGR was based only on birth weight (below the tenth percentile) in this study [35], the comprehensive molecular approach including pathway analyses is very promising, and a similar approach in a more accurately defined IUGR cohort would be highly desirable.

### 3.3. Maternal and Neonatal Serum Biomarkers

Comprehensive analyses of serum biomarkers are becoming increasingly important in a variety of diseases. As for IUGR, a cohort study demonstrated that the combined use of Doppler measurements and metabolomics from maternal serum could improve diagnostic accuracy [36]. However, it is not yet recommended to use biomarkers alone as the only diagnostic markers for IUGR. The most interesting biomarkers at present are discussed below.

In 2020, Paules et al. [37] reported proteomic profiling from maternal EDTA blood samples of pregnancies with late-onset IUGR. Among others, adiponectin was downregulated in IUGR cases, along with other dysregulated proteins affecting lipid metabolism [38]. In maternal blood samples collected between 26 and 37+6 weeks of gestation, Grohmann et al. found lower elaidic acid and gamma-linolenic acid concentrations and higher palmitoleic acid concentrations in IUGR fetuses compared to AGA fetuses. While IUGR was accurately diagnosed according to the Delphi criteria, the authors did not further differentiate the results of early and late-onset IUGR [39].

The group of Tong et al. found that the concentrations of several mRNAs, including the mRNAs *NR4A2* encoding nuclear receptor related 1 protein (NURR1) and *EMP1* encoding epithelial membrane protein 1 (EMP1), were significantly altered in the maternal circulation in pregnancies with “complicated preterm birth” (i.e., IUGR and birth before 34 weeks gestation) [40]. Combinations of 2–3 mRNAs identified IUGR particularly well, and comparable associations were present between these mRNAs and Doppler parameters, indicating placental insufficiency. Interestingly, the placenta does not appear to be the source of higher maternal circulating *NR4A2* mRNA, as the authors did not detect differential expression of *NR4A2* in placental tissue with IUGR or preeclampsia [41]. For another study, leukocytes from maternal blood during the first trimester were isolated and screened for microRNAs associated to cardiovascular diseases. The authors report that the detection rate for IUGR without preeclampsia raised about 1.52-fold compared to the sole use of routine screening criteria for preeclampsia and/or IUGR [42]. Oluklu et al. determined maternal serum midkine (neurite outgrowth-promoting factor 2) levels and found an increase in pregnancies complicated with IUGR [43]. In summary, omics approaches have detected several interesting new serum biomarkers.

While clinical studies validating the diagnostic value of micro-RNAs and lipids are still missing, the calculation of the sFLT-1/PlGF ratio is fully established and commonly used in the diagnosis of preeclampsia [44]. The angiogenetic factors sFLT-1 and PlGF are released from the placenta into maternal circulation and can easily be measured in maternal blood samples. In cases of IUGR without preeclampsia, elevated sFLT-1/PlGF ratios have also been detected [45]. It has been shown that the ratio can be used to identify severity stages of IUGR in the early-onset form [46]. In addition, a high ratio is associated with adverse neonatal outcomes, such as intrauterine fetal death (IUFD) and IUGR associated with preeclampsia [47], risk of neonatal intensive care unit admissions, and preterm delivery [48]. The ratio can also be useful in predicting fetal outcomes related to the prematurity of the baby and growth-related outcomes. The higher the ratio, the higher the risk of complications for both mother and child [49]. IUGR pregnancies diagnosed according to ultrasound criteria in which mothers have a high ratio test are at increased risk of adverse fetal outcomes (POP study) [12]. The ratio may also help to distinguish healthy SGA fetuses from those with IUGR secondary to chronic “placental insufficiency”. The trial of randomized umbilical and fetal flow in the Europe-2 (TRUFFLE-2) study is currently evaluating the ratio in late IUGR [50].

Since inflammation is supposed to play a role in IUGR, Kirici et al. recently studied pro-inflammatory markers (erythrocyte sedimentation rate, sensitivity C-reactive protein, interleukin-6, and tumor necrosis factor-alpha) and found them to be increased, while the anti-inflammatory factor interleukin-10 was decreased in the maternal serum of IUGR pregnancies. Although these findings underline the presence of a proinflammatory state in IUGR pregnancies, further validating research is needed since “IUGR” was defined as the fetal weight below the tenth percentile only, the sample size was limited, and most results showed overlaps between the groups [51].

As the most interesting approach, there is a growing number of studies measuring molecules indicative of reactive oxygen species (ROS) production in the maternal and fetal circulation. Of note, both low maternal antioxidants as well as increased amounts of ROS have been found in IUGR pregnancies [52,53,54,55]. Special interest has been directed towards thiols, ischemia modified albumin levels, Sestrin-2, and disheveled associated activator of morphogenesis 2 (DAAM2).

Free thiol groups are part of the innate antioxidant capacity, scavenging ROS [56]. Clinical research has shown that levels of free thiols in maternal serum are decreased in pregnancies with IUGR and preeclampsia, indicating decreased antioxidant capacity. However, values overlapped between the groups, limiting their diagnostic applicability [57,58,59]. Ischemia modified albumin (IMA) has especially been studied in relation to preeclampsia [60]. IMA is thought to be created when ROS causes a site-specific alteration of albumin through hydroxyl free radicals [61,62]. IMA is, indeed, significantly increased in the fetal cord blood of IUGR neonates. However, it is not elevated in maternal blood, thereby, again, confirming the increased oxidative stress in IUGR neonates as a relevant pathophysiologic mechanism but excluding IMA as a suitable maternal biomarker [63]. Expression of the Sestrin-2 gene (*SESN2*) is activated under conditions of high oxidative stress. Sestrin-2 reduces consecutive damage by directly scavenging ROS and increasing the expression of genes encoding antioxidative factors. Increased Sestrin-2 in maternal serum has been associated with IUGR and adverse neonatal outcome. A cut-off concentration to identify IUGR has also been proposed for this biomarker, but the AUC of 0.719 was only borderline [64,65]. Finally, *DAAM2* was identified by next-generation sequencing of circulating cell free RNA in pregnant women and shown to be associated with placental insufficiency, preterm IUGR, and fetal hypoxia [40]. Besides its increased concentration in maternal serum during pregnancies with growth restriction, a follow-up study showed *DAAM2* to be expressed in the placenta throughout pregnancy and to be dysregulated under growth-restricted conditions, such as hypoxia [66]. In summary, the identification and characterization of these biomarkers are promising advances for early diagnosis of IUGR. However, their diagnostic sensitivity and specificity still need to be addressed in large clinical trials.

## 4. Comprehensive Molecular Analyses Identify Oxidative Stress as a Key Mechanism in IUGR

Most of the knowledge about the potential molecular mechanisms of IUGR in humans is based on animal studies. However, the results of animal experiments cannot always be transferred to humans. Excitingly, comprehensive molecular analyses from small amounts of human material have become much more affordable in the last decade. Therefore, it is increasingly feasible to elucidate the molecular basis of health and disease from human studies.

Omics techniques in accurately defined human IUGR cohorts offer the possibility of gaining important new insights into the pathophysiology of IUGR, as they allow a comprehensive identification and quantification of differentially expressed gene transcripts, proteins, lipids, or other metabolites. During a routine follow-up of pregnant women or around birth, various biosamples can be collected non-invasively. Recent human studies have been performed in placenta [67,68,69], maternal [37,70,71,72] or umbilical cord blood [70,71], and maternal feces [72]. Multi-omics analyses help to develop hypotheses about the pathophysiological alterations occurring in the IUGR mother and placenta, as well as the adaptions that take place in the IUGR fetus. As discussed above, biomarker studies indicate a key role of ROS in IUGR pregnancies. In the following, we will therefore focus on the role of oxidative stress and discuss consecutive pro-inflammatory and metabolic changes.

### 4.1. Oxidative Stress in IUGR

**Figure 2:**
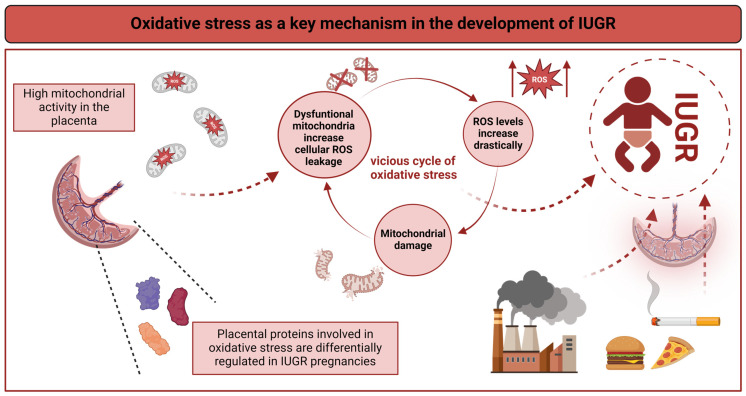
During pregnancy, oxidative stress is a key molecular (patho)mechanism in the mother, fetus, and placenta. Environmental factors such as smoking, air pollution, diet, and others may exacerbate oxidative stress. High mitochondrial activity leads to endogenous ROS production. Mitochondrial dysfunction promotes intracellular leakage of ROS. Oxidative stress, in turn, contributes to mitochondrial dysfunction, DNA damage, genomic instability, and altered cell homeostasis and survival. This creates a vicious circle of oxidative stress, which ultimately leads to inflammation and IUGR. Figure created with BioRender.com.

Oxidative stress, caused by an increase of reactive oxygen species (ROS) and/or a lack of antioxidant availability and activity, has long been linked to IUGR. Both in cases of IUGR with and without abnormal Doppler findings, e.g., due to maternal malnutrition, maternal and neonatal plasma concentrations of antioxidants have been shown to be relatively low, whilst oxidant concentrations are relatively high (Figure 2) [55,73,74]. Oxidative stress is generally high in the placenta due to its high mitochondrial activity, which leads to endogenous ROS production. Placental proteomics uncovered that a large amount of the proteins differentially expressed in placentas of late-onset IUGR pregnancies are involved in the oxidative stress response [67]. Oxidative stress even appears to precede clinical features, as an increase in maternal urinary 8-oxo-7,8-dihydro-2′-deoxyguanosine (8-oxodG) concentrations at 12 weeks gestation was associated with an increased risk for giving birth to an infant with a birth weight below the tenth percentile [75]. More recently, a cohort study comparing term and preterm SGA infants (defined as birth weight < −2 SD for gestational age) with their controls at birth showed increased serum levels of reactive oxidative metabolites in SGA infants. Moreover, the amount of oxidative stress was inversely correlated with the severity of growth restriction [76].

The exact origin of oxidative stress in the placenta remains unknown. It is thought to be largely due to inadequate perfusion and metabolic disorders, for which the evidence was well reviewed in several articles by the group of Leslie Myatt [77,78,79]. The presence of oxidative stress has been documented in various cell types involved in pregnancy complications, with recent research also showing increased oxidative stress and dysregulated homeostasis of maternal platelet cells [80]. On the molecular level, nuclear factor erythroid 2-related factor 2 (NRF2), a transcription factor with a central role in the oxidative stress response through the activation of antioxidant and oxidative damage repair agents, was shown to be differentially regulated in preeclampsia with and without IUGR [81]. This might explain previous reports on the upregulation as well as downregulation of NRF2 expression in preeclampsia and IUGR. Although IUGR is, in many cases, linked to placental pathologies such as preeclampsia, these studies suggest different IUGR ”endotypes”. Studies in this field are further complicated by the interaction of the different tissues involved (i.e., fetal, maternal, and placental structures).

In addition, environmental factors may aggravate oxidative stress. It is well known that smoking is a major risk factor for the development of IUGR. Recently, it has been shown that altered antioxidant defense mechanisms might contribute to this observation [82]. Similarly, air pollution has been shown to induce oxidative stress in the placenta and alter placental function (Figure 2) [83].

### 4.2. Oxidative Stress and Cellular Homeostasis

While the cascade leading to the final accumulation of oxidative stress end products has not yet been fully elucidated, there are several well-known sequelae. Oxidative stress causes DNA damage, reduces the genomic stability, and alters cell homeostasis and survival.

Genomic instability is one of the nine hallmarks of aging [84] and not the only one to be found in placental pathologies. A second hallmark is reduced telomere length, which has been shown in the placental tissue of IUGR pregnancies [85]. Third, mitochondrial dysfunction plays a role. Mitochondrial swelling in endothelial cells has been shown in preeclampsia and IUGR-derived cells in vitro [86]. In addition, mitochondrial dysfunction has been shown in IUGR fetuses in several animal models, as reviewed by Pendleton et al., visualizing the consequences of placental dysfunction for the fetus [87]. Mitochondrial dysfunction, in turn, leads to intracellular leakage of ROS, thus indicating a vicious circle of oxidative stress (Figure 2). Furthermore, byproducts of lipid peroxidation have been found to induce senescence in placental cells of preeclamptic women [88]. While cellular senescence is a physiological feature at the end of the placental life span, recent studies have shown that cellular senescence is accelerated in IUGR and unexplained cases of term stillbirth [89]. Taken together, these findings indicate that oxidative stress related with IUGR can trigger aging-associated processes and adversely affect cellular homeostasis as well as survival.

### 4.3. Oxidative Stress and Inflammation: A Vicious Cycle

In a recent study, human placental explants from healthy pregnancies were collected at term and exposed to different stress conditions, including hypoxia (1% oxygen) and oxidative stress (1 mM H_2_O_2_). Although hypoxia and oxidative stress differentially affected the expression and release of damage-associated molecular patterns (DAMPs) and cytokines [90], both conditions induced a pro-inflammatory profile.

These findings solidified that changes in oxygen concentration can induce sterile placental inflammation, possibly aggravating placental dysfunction and disturbing fetal development in IUGR pregnancy. Recently, the NLRP3 inflammasome was identified as a central pro-inflammatory regulator both in IUGR placental tissue as well as in a mouse model of IUGR [69]. However, in placental explants of preeclamptic pregnancies, NLRP3 inflammasome activation and IL-1β expression were significantly increased in the subgroup without IUGR only [91]. In another study, metabolomics were performed in maternal and fetal plasma at delivery. Interestingly, a pro-inflammatory profile could be shown in IUGR fetuses with or without maternal preeclampsia but was more pronounced in IUGR with preeclampsia [71].

## 5. Follow-Up Care of Children “at Risk” after IUGR Using the Example of Neuropediatric Sequelae

Epidemiological research has revealed that IUGR may have sequelae for later health and disease in almost any organ system. A considerable amount of literature has been published on this association, although there are few studies with strict IUGR criteria [92]. What needs to be kept in mind is that the adult clinical phenotype of an IUGR born individual is not “fixed” at birth [93]. Therefore, clinical follow-up strategies are needed to identify children “at risk” for adverse sequelae at an early stage in order to enable early intervention and individual support strategies to improve developmental trajectories and ameliorate adverse long-term consequences [94]. In the following, we will discuss this need, looking at the neuropediatric sequelae of IUGR-born infants.

Although IUGR children have a higher risk of neurodevelopmental impairment, individual neurocognitive outcomes may vary widely. Sacchi and colleagues performed a systematic review of 60 studies including 52,822 children and found significantly impaired cognitive outcomes in children born with IUGR, affecting both preterm and term infants [95]. Benitez et al. used the Battelle Developmental Inventory (BDI) to assess the developmental progress of 70 children with IUGR at an age of 6 years. A significant proportion (57.1%) exhibited delays across multiple development domains. Motor and communication skills were most commonly affected [96]. However, when evaluating neurodevelopmental outcomes, it is critical to distinguish between SGA and IUGR, as stated above. Therefore, Moneith et al. compared developmental outcomes of children born SGA and those born with IUGR. A total of 375 children, who were either SGA or had experienced IUGR, were assessed at 3 years of age using the Bayley Scales of Infant Development, the third edition of the Toddler Development test and the Age and Stage Questionnaire. Children from IUGR pregnancies demonstrated a poorer neurodevelopmental outcome compared to their SGA counterparts [97]. Furthermore, within the IUGR group, children with an abnormal cerebroplacental ratio had a poorer neurodevelopmental outcome than children born from pregnancies with abnormal umbilical artery Doppler evidence alone [97].

Postnatally, cerebral ultrasound sonography is commonly used to monitor brain development in the first few weeks of life after IUGR. A recent study by Aisa and colleagues suggests the additional use of 3D ultrasound sonography to detect changes in brain volume as an early indicator of neurodevelopmental disorders [98]. Further studies on changes of cerebral morphology after IUGR have been reviewed by Dudink et al. [99]. Thus, IUGR has been associated with reduced total brain volume and significant alterations of gray and white matter in the brain [99]. Recently, this was confirmed in a magnetic resonance imaging study by Korkalainen and colleagues in 32 children aged 8 to 10 years after IUGR birth. IUGR children had smaller total intracranial volumes compared to children with normal fetal growth. While the authors could not find significant volume differences in gray and white matter, IUGR was associated with alterations of white matter microstructure [100].

So far, guidelines on follow-up care after IUGR are sparse. There is no consent as to early screenings, intervention, and support services. Considering the significantly increased risk of neurodevelopmental impairment but also other organ sequelae, strategies to detect “early warning signs” exceeding the standard follow-up of child development would be highly desirable. In our institution, we are therefore evaluating the establishment of regular interdisciplinary aftercare in premature IUGR babies modelled on neurological aftercare timepoints for premature babies.

## 6. Strategies to Prevent or Mitigate IUGR

### 6.1. The Clinical View—Current Clinical Practice

Ideally, all women should receive detailed counseling before conception, including on the risk of recurrence of IUGR (23% in women with previous IUGR vs. 3% in women without previous IUGR) [101]. Furthermore, all pregnant women should be examined for possible maternal, fetal, and utero-placental risk factors for the occurrence of IUGR as part of a detailed medical history.

In principle, all pregnant women should also be advised to abstain from nicotine. If there is an increased risk of uteroplacental dysfunction with the risk of IUGR, low-dose acetylsalicylic acid (ASA; 100–150 mg/die) should be started prophylactically at < 16 weeks gestation [102]. Patients with antiphospholipid syndrome (APS) should be advised before conception and treated with low-dose ASA and low-molecular-weight heparin from early pregnancy onwards [103]. Nutritional interventions supplementing the nitric oxide (NO) precursor levo-arginine had beneficial effects on fetoplacental circulation, birth weight, and neonatal outcomes [104]. In a pilot-study in pregnancies at risk for IUGR, preterm birth, and/or preeclampsia, the NO-donor pentaerytrithyltetranitrate (PETN) decreased the risk for IUGR, perinatal death, and preterm birth and improved Doppler findings [105,106].

Life style interventions including healthy diet and appropriate daily physical activity support maternal cardiac output and circulation [107]. In contrast, maternal adiposity increases the frequency and severity of IUGR and should be avoided [108]. Recommendations exist for body weight before conception, weight gain during gestation, intake of macronutrients, and supplementation of folic acid, iodine, iron, and docosahexaenoic acid (DHA) [109].

### 6.2. The Research View—Future Perspectives

Since oxidative stress has increasingly been recognized as a major pathomechanism in the development of IUGR, the question of appropriate prevention approaches arises. Knowledge transfer within the general population on detrimental effects of smoking [82] or air pollution [83] on fetal development remains of major importance. In addition, intervention strategies aiming to reduce oxidative stress whenever it is reaching a pathological threshold have been studied. A recent meta-analysis has evaluated the effect of sildenafil citrate on pregnancy outcomes in women with IUGR. Nine studies were included, which considerably differed in terms of dosage and duration of therapy. Increased birth weight and prolonged pregnancies after sildenafil were found, but there was no positive effect on outcome parameters, including rates of stillbirth, neonatal deaths, and neonatal intensive care unit admissions [110]. In the largest study included in the meta-analysis, sildenafil failed to reduce perinatal mortality and morbidity but increased the risk of pulmonary hypertension in neonates [111]. Thus, there is no recommendation for the use of sildenafil in the societies’ guidelines.

Another large meta-analysis has recently shown that antioxidant therapy might reduce the risk of IUGR when administered after diagnosis of preeclampsia [112]. However, the studies included in this analysis were very heterogeneous using different antioxidant compounds. In addition, the studies partially contradict each other, with some showing a beneficial effect of one substance, whilst others show no effect of the same substance. Similarly, although 10 weeks of supplementation with the anti-inflammatory and antioxidant zinc improved the total antioxidant capacity and reduced CRP as an inflammatory parameter, it did not improve the pathological Doppler waves in pregnancies at risk for IUGR [113]. On a positive note, docosahexaenoic acid (DHA) has been shown to have an anti-inflammatory effect in LPS-induced placental inflammation and to reduce IUGR [114]. Evidence suggests that antioxidant properties of DHA are mainly based on its ability to enhance mitochondrial functions and biogenesis [115]. In conclusion, antioxidants might be a therapeutic option to avoid oxidative stress in pregnancy, but the topic needs further study to allow for successful implementation in the clinical setting. So far, no consensus has been reached yet as to the properties, timing, and dosage of antioxidant therapy.

Beyond antioxidants, there are studies on human umbilical mesenchymal stem cells (hUMSCs) in a therapeutic setting. It has been shown that preeclampsia pregnancy-derived hUMSCs produce more nitric oxide than healthy hUMSCs, thus increasing endogenous oxidative stress levels [116]. Interestingly, in a rat model for preeclampsia, it could be shown that administrating MSCs from healthy pregnant controls has a beneficial effect on overall fetal growth and inflammation [117]. This creates an exciting avenue of treatment options that is waiting to be further studied.

## 7. Summary and Future Perspectives

Up to 70% of fetuses below the tenth percentile are constitutional SGA and have a normal perinatal outcome, whereas IUGR, in particular, is associated with greater perinatal morbidity, prematurity, and mortality, as well as an increased risk for diseases in later life. Therefore, it is essential to differentiate between IUGR and SGA whenever possible. IUGR can be identified by additional abnormalities (lack of growth in the interval, pathological Doppler sonography, oligohydramnion, estimated weight < third percentile). In addition, even fetuses with an estimated weight above the tenth percentile may not have fulfilled their genetically predetermined growth potential due to adverse intrauterine conditions and have IUGR. There are also IUGR neonates incorrectly classified as SGA because of normal Doppler findings, particularly in late-onset IUGR. Unfortunately, intrauterine growth trajectories are sometimes not taken into account or cannot be assessed due to lacking data during and/or in late pregnancy. Moreover, in some cases, inappropriately slow growth cannot be detected at all with the usual biophysical diagnostic tools. Various markers have been associated with an existing IUGR. In clinical routine, however, only the Doppler sonographic examination of the fetal vessels and the determination of the sFlt-1/PlGF ratio have been established to date. The latter is used, in particular, for early-onset IUGR associated with hypertensive disorders of pregnancy. Recent studies have focused on oxidative stress as an important pathomechanism in SGA/IUGR and identified new candidates that could be suitable as biomarkers. Further research in accurately defined human IUGR cohorts is needed to further increase diagnostic accuracy and eventually transfer these approaches into clinical routine. The application of “omics” techniques in these cohorts offers the opportunity of gaining important new insights into the pathophysiology of IUGR, which may help to establish comprehensive molecular marker patterns, as well as new preventive and therapeutic approaches. As for IUGR prevention strategies, there is little to do beyond counseling and the optimization of life style, including diet and physical activity so far. If there is a corresponding risk of placental insufficiency, ASA should be used preventively.

In summary, recognizing fetuses and newborns being “at risk” in order to monitor them accordingly and deliver them in good time as well as to provide adequate follow up care to ameliorate adverse sequelae is still challenging, not least because diagnostic criteria still contain inconsistencies. And even though epidemiological research has clearly shown that being born after IUGR may have consequences for later health and disease in almost all organ systems, guidelines still focus on intrauterine or early postnatal morbidities and complications but do not sufficiently consider long-term sequelae. Healthcare professionals and parents still lack information on the relevance of an IUGR diagnosis, the challenges that IUGR children might face as they grow, and potential measures during development and the further course of life.

In conclusion, it would be very desirable to have more established and practicable markers available for a valid assessment of the prenatal situation and the risk of later health consequences. Both research on the pathomechanistic sequences and studies from the field of health services research on potential benefits of structured interdisciplinary follow-up care are needed to optimize individual health trajectories and avoid secondary hits after IUGR. Most importantly, awareness needs to be raised that pre- and postnatal care of IUGR neonates should be regarded as a continuum.
